# Condition-dependent trade-offs between sexual traits, body condition and immunity: the effect of novel habitats

**DOI:** 10.1186/s12862-016-0706-0

**Published:** 2016-06-21

**Authors:** Maider Iglesias-Carrasco, Megan L. Head, Michael D. Jennions, Carlos Cabido

**Affiliations:** Department of Evolutionary Ecology, National Museum of Natural Sciences, Spanish National Research Council (CSIC), José Gutiérrez Abascal, 2, 28006 Madrid, Spain; Department of Herpetology, Aranzadi Science Society, Alto de Zorroaga, 1, 20014 Donostia-San Sebastián, Spain; Division of Evolution, Ecology and Genetics, Research School of Biology, Australian National University, Canberra, ACT Australia

**Keywords:** Novel habitats, *Lissotriton helveticus*, Sexual selection, PHA, Resource allocation

## Abstract

**Background:**

The optimal allocation of resources to sexual signals and other life history traits is usually dependent on an individual’s condition, while variation in the expression of sexual traits across environments depends on the combined effects of local adaptation, mean condition, and phenotypic responses to environment-specific cues that affect resource allocation. A clear contrast can often be drawn between natural habitats and novel habitats, such as forest plantations and urban areas. In some species, males seem to change their sexual signals in these novel environments, but why this occurs and how it affects signal reliability is still poorly understood.

**Results:**

The relative size of sexual traits and level of immune responses were significantly lower for male palmate newts *Lissotriton helveticus* caught in pine and eucalyptus plantations compared to those caught in native forests, but there was no habitat-dependent difference in body condition (*n* = 18 sites, 382 males). The reliability with which sexual traits signalled body condition and immune responses was the same in all three habitats. Finally, we conducted a mesocosm experiment in which males were maintained in pine, eucalypt or oak infused water for 21 days. Males in plantation-like water (pine or eucalypt) showed significantly lower immune responses but no change in body condition. This matches the pattern seen for field-caught males. Unlike field-caught males, however, there was no relationship between water type and relative sexual trait size.

**Conclusions:**

Pine and eucalyptus plantations are likely to be detrimental to male palmate newt because they are associated with reduced immune function and smaller sexual traits. This could be because ecological aspects of these novel habitats, such as high water turbidity or changes in male-male competition, drive selection for reduced investment into sexual traits. However, it is more probable that there are differences in the ease of acquisition, hence optimal allocation, of resources among habitats. Our mesocosm experiment also provides some evidence that water toxicity is a causal factor. Our findings offer insights into how plantations affect amphibian life histories, and how novel habitats might generate long-term selection for new resource allocation strategies in native species.

**Electronic supplementary material:**

The online version of this article (doi:10.1186/s12862-016-0706-0) contains supplementary material, which is available to authorized users.

## Background

Sexual selection drives the evolution of elaborate male traits that increase mating and/or fertilization success [1]. However, these sexually selected traits can also be costly, and reduce other fitness components [2]. For example, sexual traits can decrease the ability to avoid predators, increase energy expenditure, and lower immune responses, which ultimately reduces survivorship [3, 4]. Adaptive responses to these trade-offs can occur over evolutionary time (i.e. selection for genotypes), and/or be expressed in the short-term (i.e. phenotypic plasticity).

At the individual level there is abundant evidence that condition-dependence (*sensu* [5]) drives phenotypic plasticity in the expression of sexual traits (reviews: [6, 7]). This is assumed to be adaptive because males in better condition can afford to invest more into sexual traits because they pay lower marginal costs ([8] but see [9]). Of course, the optimal expression of sexual signals also depends on the environment [10, 11]. The most intensively studied source of environment–dependent selection on sexual traits is associated with their efficacy as signals (i.e. signal-to-noise ratio for communication; e.g. [12]). However, the optimal expression of most life history traits also depends on ecological aspects of the environment. For example, the risk of mortality is partly determined by factors that affect predation risk [13], resource availability [14] and parasite loads [15]. Consequently, the survival costs of a given level of sexual trait expression, as with most life history traits, will vary across habitats [16].

Species appear to vary in whether and how they alter their sexual signals in response to variation in environmental conditions, particularly that associated with novel habitats [17]. Some species produce signals that are seemingly well adapted to new habitats [18], others do not [19], and some even respond in apparently maladaptive ways, resulting in so-called “evolutionary traps” [20, 21]. Environmental factors determine the net benefit of sexually selected traits because they alter the relative returns from investing into different fitness components. The extent of variation in sexual traits across environments will depend on the combined effects of local adaptation (i.e. genetic evolution of new allocation decisions) due to differences in mean condition (i.e. via condition-dependence) and/or changes in trade-off relationships (i.e. new cost/benefit ratios); and through adaptive phenotypic responses based on environment-specific cues that affect optimal resource allocation decisions (e.g. [22]).

Male sexual traits are often positively correlated with greater immune responses or lower parasite loads [23, 24], but see e.g. [25] and females tend to prefer males with lower parasite loads and/or greater immune function [26]. Experimental studies suggest that the immune system competes with sexually selected ornaments for resources [27–29]. This leads to a trade-off between sexual trait expression and immune function. It is well known that the observed phenotypic correlation between traits that trade-off can be positive or negative (see [30, 31] for meta-analyses of the outcome of trade-offs involving sexual traits). The sign of the relationship is usually attributed to whether there is greater variation in resource acquisition or allocation [32]. Equally importantly, however, is how the costs and benefits of sexual and other fitness-enhancing traits differ among environments. For example, immune function is less important when parasites are rare [33]; and sexual traits are less important when competition for mates is low [34], or when they are more costly to produce because males have fewer resources (i.e. poorer condition), or even when the benefit of investing in signals is reduced because the environment makes it harder to discriminate between high and low quality males [35]. The environment should therefore affect optimal investment into traits and the phenotypic relationships between these traits.

To date, few field studies have explicitly reported on the extent to which the phenotypic relationship between sexual trait expression, body condition and immune response varies among habitats [36] (but see [11] for laboratory studies). To explore this question in a focused manner it is necessary to study contrasting environments. Anthropogenically created novel habitats, such as forest plantations, provide an ideal contrast with native habitats. These are newly created, seemingly lower quality, environments that often affect individuals’ body condition and can alter the strength of sexual selection (e.g. [37–39]). Species appear to vary in whether their sexual signals change in response to novel environmental conditions [17]. To date, the effect of novel habitats on trade-offs between sexual traits and other fitness-enhancing traits, such as immune function, has been poorly explored. This is important because some studies report seemingly adaptive changes in sexual trait expression [12], which could actually be maladaptive if they shift resources away from other fitness-enhancing traits that have a greater effect on fitness in the new environment. Here we address this shortfall.

We studied the palmate newt *Lissotriton helveticus* (Razoumowsky, 1789), a common urodele in Western Europe. During the mating season males develop distinct visual secondary sexual traits, such as hind-feet webs, a caudal crest and a caudal filament (e.g. [40, 41]). These newts breed in a wide range of waterbodies (from ponds to lakes) and occur in a range of habitats, from intensively managed agricultural lands to natural forests. Among forested areas, they are most common in native forests, but are also found in exotic eucalypt and pine plantations. These plantations have been established in the study area in the last 50 years, so the evolutionary pressures faced by species inhabiting them are new. Both eucalypt and pine plantations are consistently associated with reduced species richness [42–44] and altered community structure [45]. In addition, eucalypt plantations are characterized by the release of toxic substances into the substrate [46] and waterways [47]. There is, however, little data about the effect that these habitats have on the ecology of individuals (for an exception see [48]).

We sampled males from 18 populations in pine or eucalyptus plantations, or in native oak forest. Different types of habitat patches were usually close to each other, so that gene flow due to the movement of individuals between habitats is likely. Both the mobility of newts and the short period of time that has elapsed since the establishment of plantations suggest that any differences in newt phenotypes among habitats are primarily due to plastic changes related to the characteristics of the habitat, rather than to local adaptation. If pine/eucalyptus plantations negatively affect male newts, we predict: (a) a lower expression of sexual characters, a lower immune response and poorer body condition in pine/eucalyptus plantations due to habitat-related characteristics, such as fewer food resources and/or greater toxicity; (b) a difference in the relationship between condition and immune response and/or in the extent of sexual trait development among habitats (arising from different allocation strategies). Next, we created experimental mesocosms, where we controlled food supply and manipulated the type of leaf litter (eucalypt, pine or oak leaf). We tested the extent to which observed habitat differences in male traits might be driven by changes in water toxicity that affect males during their aquatic breeding phase. If negative effects of pine/eucalyptus plantations are primarily due to leaf toxicity, we predict that (c) differences among mesocosms would mirror those seen in field-caught males.

## Methods

### Field study

**Fig. 1:**
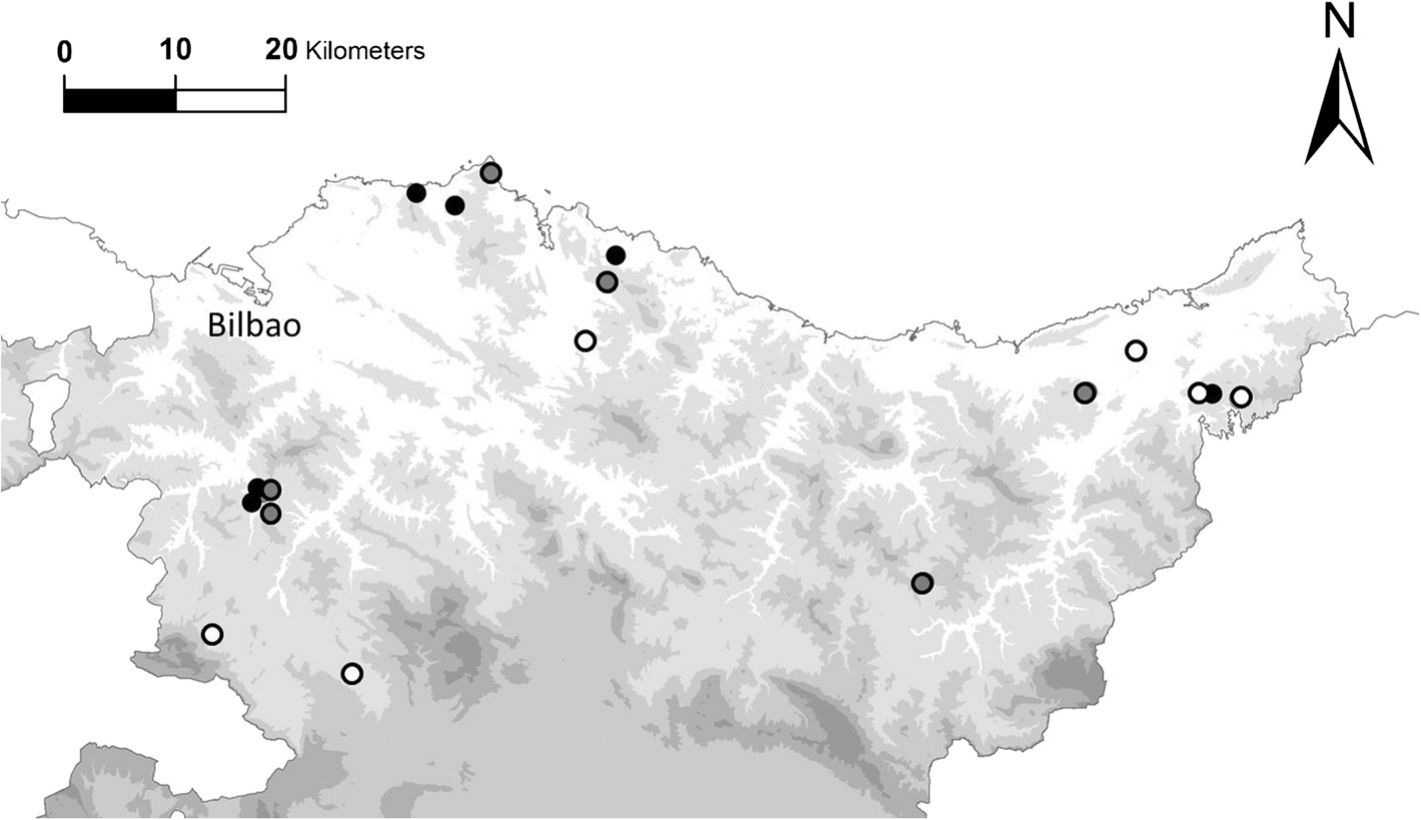
Map showing the 18 capture sites. White: natural oak forest; grey: pine plantations; black: eucalyptus plantations

From April 3–10, 2013 we captured 18–23 adult male newts from each of 18 ponds: six in native deciduous forest patches (*Quercus robur* L.), six in eucalypt plantations (*Eucalyptus globulus* Labill.) and six in pine plantations (*Pinus radiata* D.Don) in Basque Country. The vegetation in the study area is highly fragmented. The natural oak and beech forests are reduced to small patches surrounded by pine and eucalypt plantations. The ponds sampled were in habitat patches ranging in size from 0.6 to 1.2 km^2^. Forests of different types were chosen to be as close to each other as possible given the available distribution of habitats to minimize differences between populations due to microclimate characteristics of the area (i.e. precipitation, substrate, temperature; Fig. 1). The distance between the closest ponds in different habitats was between 400 and 1000 m. After an aquatic larval period of about 90 days [49], juvenile newts metamorphose and enter a long terrestrial phase (2 years), which they spend living in the forest. After this terrestrial phase the newts return to ponds as adults to breed. Once adulthood is reached, individuals usually remain within a few meters of the ponds [50], but they can also disperse several kilometres [49], a sufficient distance to readily move between sampled ponds and to colonize new habitats. All ponds were small (ranging from 2.4 to 10.7 m^2^), temporary and situated in the middle of the woodland.

We measured each male’s body condition, immune function, four sexually dimorphic traits and total body length (*n* = 382). We calculated body condition as the residuals from the regression of body mass (g) on total length (mm), both variables were first Box-Cox transformed. When the relationship between these variables is linear (as it was here: field data *p* < 0.001, *r*^2^ = 0.48; mesocosms data *p* < 0.001, *r*^2^ = 0.51) this measure is often used as an index of the relative amount of fat stored, and, hence, of nutritional status (reviewed in [51]). It is possible that the body mass of newts is related to the amount of food they have recently eaten and hence, in part reflects gut load. To counter this concern we reduced the influence of recent feeding on body mass measures by waiting 16 h after capture before weighing animals. The regression of body mass on body length is commonly used in newts as a non-invasive method to measure condition, and has successfully been used to investigate differences in food availability and habitat quality [52, 53].

To measure immune function we used a phytohaemagglutinin injection assay (PHA test), which is a delayed-type hypersensitivity test. This test is a reliable measure of T-cell dependent immunocompetence in vivo [54], and has been used and validated in many studies including those on amphibians [55, 56] (Note, however, that it does not capture all aspects of immune function: see [57]). We measured the thickness of the base of the tail 5 mm from the vent with a pressure-sensitive spessimeter (+0.01 mm). The spessimeter closes to the point at which it touches the skin of the animal. It is important not to press the skin, because the inflammation caused by the PHA can disappear with the pressure of the spessimeter and lead to an incorrect post-injection measure. Our measurements were highly repeatable based on being taken five times. We used the average measurement for our statistical analyses. Immediately after the initial measurements, we then injected males with 0.01 mg of PHA dissolved in 0.01 ml of phosphate-buffered saline (PBS). All individuals were first anaesthetized by immersion in Tricaine methane sulphonate (0,15 g MS-222/1 L dechlorinated water) for 5–10 min [58]. Once recovered post-injection, newts were placed into plastic containers with 1 L of dechlorinated water. After 24 h, we measured the thickness of the tail base at the same point to calculate the difference between pre- and post-injection measures (inflammation). The cellular immune response index (hereafter ‘PHA immune response’) was calculated as the residuals of the regression of the inflammation against snout-vent length (all variables Box-cox transformed; [54, 59]). The only appreciable effect of the PHA injection was a slight swelling of the skin, caused by the immune response, which disappeared after 48 h. None of the newts showed any sign of stress or pain during these tests, and all looked healthy after the trials. All newts were fed after the second measure and were returned to their capture sites 48 h after being captured.

Male *Lissotriton helveticus* have several secondary sexual morphological characters that are involved in female mate choice: hind feet webs, caudal crests and caudal filaments (e.g. [40, 41]). Body dimensions were taken from photos of anaesthetized newts placed on a glass board with a measurement scale. We measured four secondary sex characters (caudal crest area, caudal crest depth, caudal filament length and hind feet webbing) and total body length. After the second measurement for the PHA test, animals were maintained in water to allow them to freely open their hind feet webs and another photo was taken from above to measure hind feet web area. All measurements were made using Image J [60].

### Mesocosm study

We had three experimental treatments (oak, pine or eucalyptus leaves) with three replicates of each. To simulate natural pond conditions we used 470 L mesocosms containing 200 L of tap water. Extracts were prepared by placing 100 g of dried leaves in each mesocosm. We also added 5 L of natural pond water from natural oak forest containing zooplankton and algae. The mesocosms were covered with a mesh to prevent the entry of predators. Then, after 48 h, we captured 180 adult male palmate newts from three nearby natural ponds in a mixed beech-oak natural forest. We randomly assigned 20 males to each of the 9 mesocosms, along with five females per mesocosm so that males stayed in the mesocosm, and maintained their secondary sexual characters (i.e. did not enter the terrestrial phase because of a lack of mating possibilities). Every day 5 g of blood worms were added to each mesocosm. We kept the animals in the mesocosms for 21 days to look for short-term responses to our treatments. Based on previous observations in the lab, the sexual traits of males become significantly reduced after a few days in captivity, presumably due to the initial stress (ICM personal observation). Moreover, 21 days is similar to the period of exposure used in other toxicological assays of amphibians [61]. Thereafter all animals were individually placed in aquaria with 1 L of clean water to take the same morphological and immune measurements described for the field study. The individuals used for the mesocosms study were captured in three adjacent ponds, between which individuals were likely to have free movement. Afterwards, animals were returned to the capture sites.

### Statistical analyses

The measured traits (PHA immune response, body condition; and four sexual traits: tail crest depth, caudal filament, tail crest area and hind-feet web) were Box-Cox transformed to better meet assumptions of normality. We then calculated the residuals for each sexual trait from its regression on snout vent length. To reduce the number of independent variables for sexual traits (and decrease potential problems associated with multiple testing), we ran a Principal Component Analysis on these residuals. The residuals of all four sexual traits loaded strongly and positively on PC1, which explained 71 % of the variance for the field data and 59 % of the variance for the mesocosms. PC1 was used as our measure of the relative expression of sexual traits (hereafter ‘relative sexual trait size’). It is, however, possible that the different sexual signals measured are differentially influenced by habitat type and/or condition. To explore this possibility we repeated all the models for each trait separately (*post hoc* test results are presented as Additional file 1). The statistical analyses and results presented in the main text are based on PC1 of all four sexual traits.

We used Linear Mixed Models (LMM) to test for the effect of habitat type on our male measurements. Models were run in R 3.2.2, or with Statistica 13.0. We treated replicate (i.e. forest) as a random factor, and habitat as a fixed effect. We conducted post-hoc pairwise comparison using Tukey tests. We checked the residuals of all models using q-q plots to ensure that they met the assumption of normality, linearity and homoscedasticity.

To test whether the relationship between relative sexual trait size and PHA or body condition respectively differed among the three forest types, we ran separate LMMs with PHA response or body condition as the dependent variables, and relative sexual trait size, and forest type as fixed effects. We first standardized relative sexual trait size to a mean of zero and a standard deviation of one to assist in interpretation of the results [62].

We repeated the same statistical analyses for the effect of experimental treatment in the mesocosm study.

## Results

### Field experiment

Summary statistics for each forest type are presented in Table 1. Relative sexual trait size differed significantly among the three forest types (*F*_2,15.06_ = 23.25, *P* < 0.001, Table 1a). Sexual trait expression was highest in newts from oak forest, intermediate in pine plantations and much lower in those from eucalyptus plantations (all pairwise tests, *p* < 0.001). We also found significant habitat differences in male immune response to PHA (*F*_2,14.78_ = 25.57, *P* < 0.001, Table 1a). The PHA immune response of males from oak forest was much higher than that of males inhabiting pine or eucalyptus plantations (both pairwise tests, *p* < 0.001). PHA immune response was also higher in pine than eucalyptus plantations (pairwise test *p* < 0.001). Surprisingly, however, male body condition did not differ among the three habitats (*F*_2,15.19_ = 1.90, *P* = 0.183, Table 1a).

**Table 1:** Differences in the relative sexual traits, PHA response and body condition among habitats in a) field data and b) mesocosms

	Mean ± SD				
Trait	Oak	Pine	Eucalyptus	F	*P* - value
a) Field					
Relative sexual traits	0.740 ± 0.76	−0.245 ± 0.82	−0.742 ± 0.75	23.25	**<0.001**
PHA response	0.034 ± 0.03	0.002 ± 0.04	−0.050 ± 0.03	25.57	**<0.001**
Body condition	0.002 ± 0.02	0.007 ± 0.02	−0.011 ± 0.02	1.9	0.18
Total length (cm)	6.804 ± 0.45	7.036 ± 0.43	7.013 + 0.38	1.92	0.18
Weight (g)	1.468 ± 0.24	1.587 ± 0.24	1.468 ± 0.24	1.25	0.31
b) Mesocosms					
Relative sexual traits	0.303 ± 0.97	−0.187 ± 0.80	−0.117 ± 1.14	2.52	0.16
PHA response	0.242 ± 0.21	0.018 ± 0.22	−0.265 ± 0.22	29.65	**<0.001**
Body condition	−0.006 ± 0.11	−0.004 ± 0.14	0.010 ± 0.13	1.29	0.275

Significant values are in bold. Sample sizes in text

**Fig. 2:**
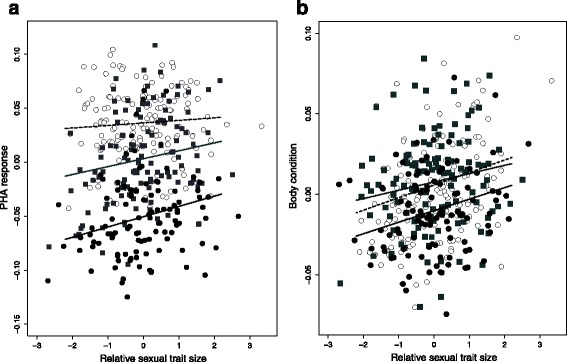
The relationship between relative sexual trait expression and: (**a**) PHA immune response was positive in all three habitats, model *R* ^*2*^: 0.62; (**b**) body condition was positive in all three habitats, model *R* ^*2*^: 0.42. The strength of the relationship did not differ among forests for either PHA immune response or body condition (see main text). Oak forest: white circles, dashed line; pine plantations: grey squares, grey solid line; eucalyptus plantations: black circles, black solid line

Males with larger sexual ornaments for their body size had stronger PHA immune responses in all three habitats (PC1: estimate ± SE: 0.012 ± 0.004; *t*_*369.8*_ = 2.945, *P* = 0.003, Fig. 2a), and the strength of the relationship did not differ among the three forest types (interaction: estimate ± SE: 0.005 ± 2.1e ^−5^; *t*_*373.8*_ = 0.985, *P* = 0.25; Fig. 2a). Similarly, males with larger sexual ornaments for their body size were in better body condition (estimate ± SE: 0.009 ± 0.003, *t*_*368*_ = 3.156, *P* = 0.001, Fig. 2b), but, again, there was no difference in the strength of the relationship across the three forest types (estimate ± SE: 0.003 ± 1.6 e ^−5^, *t*_*371*_ = 0.973, *P* = 0.56, Fig. 2b).

### Mesocosms experiment

There was no effect of treatment on either relative sexual trait size or body condition when males were maintained for 21 days in mesocosms (sexual traits: *F*_2,5.97_ = 2.52, *P* = 0.160; body condition: *F*_2,179_ = 1.29, *P* = 0.275; Table 1b). However, this short period of time was sufficient to affect male PHA immune response (*F*_2,6.12_ = 29.65, *P* < 0.001; Table 1b). As with field-caught males the PHA immune response was highest in the oak treatment males, intermediate in pine treatment males and lowest in males maintained in eucalyptus leaf litter (all pairwise tests < 0.01). Summary statistics are presented in Table 1b.

**Fig. 3:**
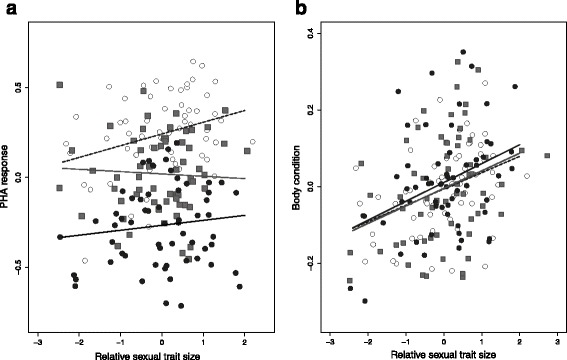
In males in mesocosms there was (**a**) no relationship between sexual trait expression and PHA immune response in any of the three treatments, model *R* ^*2*^: 0.51; (**b**) a significant positive relationship with body condition that did not differ in strength among the three treatments (see text), model *R* ^*2*^ : 0.12. Oak forest: white circles, dashed line; pine plantations: grey squares, grey solid line; eucalyptus plantations: black circles, black solid line

Unlike the case for field caught males, there was no relationship between sexual trait expression and PHA immune response (estimate ± SE: 0.024 ± 0.024, *t*_*177.1*_ = 0.978, *P* = 0.328; Fig. 3a). There was, however, still a positive relationship between sexual trait expression and body condition (estimate ± SE: 0.042 ± 0.013, *t*_*179*_ = 3.120, *P* = 0.002; Fig. 3b), and, as in the field collected males, the strength of the relationship did not differ among treatments (estimate ± SE: −0.013 ± 0.002, *t*_*179*_ = −0.553, *P* = 0.374; Fig. 3b).

In general, we observed the same general pattern seen for PC1 (i.e. relative sexual trait size) when we ran the models separately for the relative size of each sexual trait (Additional file 1). However, we found slight differences among traits when looking at the relationship between each trait and body condition or PHA response (Additional file 1: Table S2 and S3). These findings suggest that some traits might be less costly to produce and, hence, less reliable indicators of male quality. These results are a reminder of the importance of studying several traits when assessing the effect of habitat alteration on male sexual signaling.

## Discussion

We predicted that inhabiting novel forest plantations would change the resource acquisition of male palmate newts (*Lissotriton helveticus*) due to specific characteristics of these habitats (e.g. reduction of food both in the aquatic and terrestrial phases [43, 44], or confronting toxic substances [63]) and that this would reduce the amount of resources that males can invest in traits that are sexually selected and traits that enhance viability. In support, we found a significant reduction in relative sexual trait size and immune response in wild caught males from Eucalyptus or pine plantations compared to those from natural forests. There was, however, no effect of forest type on male body condition. We did not find differences among habitats in the relationship between sexual traits and either body condition or immune response. These findings suggest that the reliability of male sexual ornaments as signals does not change and that they are good predictors of immune response and body condition in both native and novel habitats. Finally, the similarities between the field and mesocosm data show that water toxicity might play an important role in the observed habitat differences. Although palmate newts can inhabit forest plantations these habitats seem to have a harmful effect (inferred from smaller sexual traits and weaker immune responses), which could affect the evolution of reproductive strategies (e.g. resource allocation, mate choice) in the long-term.

### Mean trait values

The lack of a detectable difference in body condition between novel and native forests seemingly contradicts our assumption that plantations are a low quality habitat. It is, however, important to distinguish between the theoretical concept of condition (i.e. total resources available *prior* to allocation to life history traits; see [5] and measured body condition (i.e. some measure of the ratio of fat or body mass to body length) (reviews: [6, 7]). Measured body condition is itself a life-history trait, and variation in body condition could adaptively vary (or remain constant) among environments depending on the relative fitness returns from investing in it as opposed to other traits (e.g. sexual traits or immune function).

Plantations could have negative effects on trait expression not only due to lower resource availability, but also due to changes in other environmental factors that select for different optima. These factors include the greater prevalence of diseases, or higher parasite loads (e.g. [64]). If so, it might be adaptive for males to invest less into sexual signals and more into immune response. Compared to males from native habitat, however, the immune response to PHA was lower for field-caught males from plantations. The same was true for males kept in mesocosms mimicking the water conditions in the respective habitats. Previous laboratory experiments show that immune function can change rapidly when exposed to toxins as a stress-induced response [65]. The allelopathic substances in eucalyptus and pine leaves might therefore directly reduce immune function. However, the observed immune response to PHA is not necessarily reflective of total investment into immune function. It has to be discounted by the extent to which resources have already been used to counter earlier infections and/or dealing with toxins (for a thorough review see [57]).

In our field data, males from pine plantations reduced their investment in both sexual traits and immune response compared to those from natural oak forests, but less so than males from eucalyptus plantations. We observed the same pattern for immune responses (PHA), but not for sexual traits, in the mesocosms where the amount of food provided was kept constant across treatments. The toxicity of eucalyptus leaves is known to have a strong effect on some animal communities [66]. If eucalypt leaves are more toxic than pine leaves we expected newts from eucalyptus plantations to invest more of their resources into trying to expel toxins from their body. If males invest resources into expelling these toxins, the resources that are then available to invest into other traits, such as sexual characters and immune response, should be reduced. The toxicity of the water, especially in the case of eucalyptus, might therefore partly explain the differences in immune response to PHA found between oak forests, pine and eucalypt plantations in both our field and mesocosms experiments. On the other hand, sexual trait expression might only change plastically over a longer time scale than the 21 days of our study, or in response to environmental characteristics not manipulated in our mesocosms study. For example, investment in sexual traits might depend on the conditions encountered on land and the resources acquired during the terrestrial phase. Males develop sexual traits only during the breeding aquatic phase, while during the terrestrial stage they have no secondary traits. In plantations, the availability of terrestrial prey seems to be lower [43] and the environmental conditions harsher (i.e. less refuge availability or presence of toxic substances), affecting the availability of resources for sexual characters upon entering the water phase. Once the investment in sexual traits is completed, even if the water conditions worsen (as in our mesocosms experiment), animals might strategically maintain their investment in sexual characters to avoid losing mating opportunities, but in so doing they might then compromise other fitness-related traits.

Differences in sexual trait expression between habitat types could also be explained by other ecological variables, such as water turbidity or male competition. For example, water turbidity is known to have a negative effect on the expression of sexual traits in *L. helveticus* [67]. In turbid environments, visual signals are less effective, so males might show an adaptive phenotypically plastic response, and reduce investment into costly visual sexual signals and reallocate resources elsewhere [38]. Social communication in newts depends on both visual and chemical signals [68, 69]. Water turbidity could reduce the advantage of developing visual sexual signals traits, so that males will benefit from greater investment into pheromones. However, the leachates released by tree plantations, can also disrupt mate choice based on chemical cues (e.g. disruption of mate choice in newts by endosulfan [70] or realistic nitrate concentrations [71]), which could have a strong effect on sexual selection in these novel habitats. Moreover, when male-male competition for females is higher we generally expect stronger investment in sexually selected traits. In another study, however, we found that neither water turbidity nor the population density differs among study ponds in different habitat types (Iglesias-Carrasco et al., unpublished).

Adaptive responses to a trade-off between elevating reproductive success and decreasing survival can occur over evolutionary time by adaptation and in the short-term by phenotypic plasticity. However, the pine and eucalyptus plantations in our study were established less than 50 years ago. Gene flow between populations in different habitats is highly likely because the populations are not isolated (there are patches of mixed plantations with natural forests throughout the region). The observed changes in trait expression in plantations are therefore most likely to be due to phenotypic plasticity and differential acquisition allocation of resources and not to genetic adaptation to the new environment.

### Relationships between traits

Environmental differences can create variation in the level of condition-dependence of sexual traits [72]. This variation arises when the optimal resolution of trade-offs, such as investing into self-maintenance or the production of costly ornaments, differs between contrasting environments [73]. This could result in the long-term evolution of population differences in allocation decisions (i.e. the relationship between condition and a given trait), as predicted by life history theory [16]. It is, however, necessary to be careful when talking about adaptive phenotypic plasticity and optimal resource allocation. Some populations and species exhibit pre-adaptations to novel environments, especially when the conditions in the original habitat are similar to those in the novel habitat [74]. Life-history strategies seem to play an important role in the capacity to invade new habitats [75]. In general, however, organisms will only show an adaptive phenotypic response in, say, sexual trait expression or immune function if the optimal allocation *and* the proximate cues for how to allocate resources are similar in both the novel and historically encountered environments (i.e. those that have shaped phenotypic plasticity to make it adaptive). It is also plausible that being in an novel habitat amplifies or reduces the relative difference in condition between low and high quality individuals (e.g. a stressful environments can similarly affect the magnitude of inbreeding depression [76, 77]). This could alter the strength, and possibly even the direction, of relationships between sexual traits and other fitness components [36, 72, 78].

In general we found that field-caught males with relatively larger sexual traits were in better body condition and had a stronger immune response to PHA. Assuming that plantations are a lower quality habitat, and given the observed lack of differences in mean body condition between plantations and native forests, we expected the phenotypic relationship between body condition, sexual trait size and immune response to differ among habitats (i.e. resources being diverted away from the latter two traits to maintain body condition in plantations). There was, however, no difference among habitats in the mean strength of the relationship for either trait (Fig. 2a, b). One explanation for this finding is that we only measured a few life history traits. The relative allocation to other unmeasured traits might change across habitats based on cues other than condition, such as perceived predation risk or food availability, so that the relative investment into sexual traits and body condition or immunity remains constant.

Stronger sexual selection for condition-dependent male sexual traits has been suggested to occur in both favorable [79] and stressful environments [80]. In contrast to other empirical studies [36], however, our findings suggest that male sexual ornaments are equally reliable predictors of immune response and body condition in both low and high quality habitats. Reliable signaling is a key concept to understand how male ornamentation has coevolved with female preferences [10]. However, due to environmental heterogeneity [10, 81], a mismatch between a male’s sexual trait expression and his underlying fitness can compromise the benefits of female mating preferences [82]. Although the reliability of male signals of specific life history traits did not change (i.e. body condition and immune response to PHA), it is unknown whether these traits are themselves equally valuable in all habitats, hence whether males are always reliably signaling their *net* fitness. In addition, we need to consider the effect of the habitat on female mate choice, which could possibly be mediated by changes in condition that affect her own mate choice decisions (e.g. [83–85]).

## Conclusion

Male *L. helveticus* sexual signals differ in mean expression between a native and two novel habitats. Despite this, the reliability of these sexual traits as signals of body condition and immune responses was the same across all three habitats. The extent to which this is maladaptive and generates selection for the evolution of new male allocation strategies will ultimately depend on the net fitness of males who continue to use allocation strategies that evolved in very different environments to these novel habitats.

## Additional file

Additional file 1: Table 1S. Differences in the relative tail crest area, tail crest depth, tail filament length and web area among habitats in a) field data and b) mesocosms. Significant values are in bold. Sample sizes for all experiments can be found in the main text. **Table S2**. The results of the models of the relationships between each relative sexual trait expression and: (a) body condition, (b) PHA response in the field data. **Table S3**. The results of the models of the relationships between each relative sexual trait expression and: (a) body condition, (b) PHA response in the mesocosms data. (DOCX 36 kb)
