# Supplementary material for: Condition-dependent trade-offs between sexual traits, body condition and immunity: the effect of novel habitats
Source: BMC Evol Biol. 2016 Jun 21;16:135. doi: 10.1186/s12862-016-0706-0 (PMC4915150; doi:10.1186/s12862-016-0706-0)
Supplement: Additional file 1: Table 1S. — Differences in the relative tail crest area, tail crest depth, tail filament length and web area among habitats in a) field data and b) mesocosms. Significant values are in bold. Sample sizes for all experiments can be found in the main text. Table S2. The results of the models of the relationships between each relative sexual trait expression and: (a) body condition, (b) PHA response in the field data. Table S3. The results of the models of the relationships between each relative sexual trait expression and: (a) body condition, (b) PHA response in the mesocosms data. (DOCX 36 kb) [file 12862_2016_706_MOESM1_ESM.docx]

Additional file 1

Table S1. Differences in the relative tail crest area, tail crest depth, tail filament length and web area among habitats in a) field data and b) mesocosms. Significant values are in bold. Sample sizes in main text.

a)

| Field data | | | | | |
| --- | --- | --- | --- | --- | --- |
|  | Mean + SD | | |  |  |
| Trait | Oak | Pine | Eucalyptus | F | P - value |
| Tail area | 0.019 + 0.03 | -0.003 + 0.034 | -0.022 + 0.03 | 10.52 | **0.001** |
| Tail depth | 0.014 + 0.019 | -0.003 + 0.023 | -0.016 + 0.01 | 22.07 | **<0.001** |
| Filament length | 0.201 + 0.039 | 0.165 + 0.038 | 0.158 + 0.36 | 7.75 | **0.004** |
| Web area | 0.585 + 0.563 | -0.232 + 0.576 | -0.544 + 0.518 | 37,57 | **< 0.001** |

b)

| Mesocosms data | | | | | |
| --- | --- | --- | --- | --- | --- |
|  | Mean + SD | | |  |  |
| Trait | Oak | Pine | Eucalyptus | F | P - value |
| Tail area | 0.051 + 0.18 | -0.059 + 0.15 | 0.007 + 0.20 | 2.26 | 0.185 |
| Tail depth | 0.018 + 0.07 | -0.008 + 0.05 | -0.010 + 0.08 | 1.82 | 0.240 |
| Filament length | 0.015 + 0.07 | -0.002 + 0.07 | -0.013 + 0.07 | 2.42 | 0.091 |
| Web area | 0.035 + 0.18 | -0.012 + 0.16 | -0.024 + 0.18 | 1.43 | 0.310 |

Table S2. The results of the models of the relationships between each relative sexual trait expression and: (a) and body condition, (b) PHA response in the field data. Sample sizes in main text.

| 1. Field data (sexual traits in relationship with body condition) | | | | | | |
| --- | --- | --- | --- | --- | --- | --- |
| Trait | Predictor | Estimate | SE | df | t-value | P - value |
| Body condition | Intercept | -0.006 | 0.007 | 17.3 | -0.810 | 0.426 |
|  | Oak forest | 0.009 | 0.01 | 16.5 | 0.910 | 0.373 |
|  | Pine forest | 0.014 | 0.01 | 16.1 | 1.358 | 0.193 |
|  | Tail area | 0.182 | 0.077 | 370.5 | 2.351 | **0.019** |
|  | Oak forest x tail area | -0.050 | 0.103 | 371.8 | -0.550 | 0.582 |
|  | Pine forest x tail area | -0.105 | 0.103 | 372.4 | -1.015 | 0.310 |
|  | Intercept | -0.004 | 0.007 | 18.2 | -0.598 | 0.557 |
|  | Oak forest | 0.003 | 0.01 | 17.3 | 0.337 | 0.74 |
|  | Pine forest | 0.012 | 0.01 | 16.6 | 1.225 | 0.237 |
|  | Tail depth | 0.352 | 0.127 | 368 | 2.755 | **0.006** |
|  | Oak forest x tail depth | 0.009 | 0.165 | 369.5 | 0.577 | 0.564 |
|  | Pine forest x tail depth | -0.255 | 0.157 | 367.9 | -1.615 | 0.107 |
|  | Intercept | -0.021 | 0.012 | 113.1 | -1.734 | 0.085 |
|  | Oak forest | 0.026 | 0.018 | 119.1 | 1.464 | 0.145 |
|  | Pine forest | 0.024 | 0.017 | 107 | 1.369 | 0.173 |
|  | Filament length | 0.073 | 0.064 | 367.7 | 1.127 | 0.26 |
|  | Oak forest x filament length | -0.069 | 0.086 | 372.8 | -0.804 | 0.422 |
|  | Pine forest x filament length | -0.039 | 0.088 | 369.5 | -0.450 | 0.653 |
|  | Intercept | -7.7e^-4^ | 0.006 | 19.2 | -0.111 | 0.913 |
|  | Oak forest | -0.004 | 0.009 | 18.5 | -0.436 | 0.668 |
|  | Pine forest | 0.012 | 0.009 | 17.3 | 1.343 | 0.197 |
|  | Web area | 0.017 | 0.004 | 264.2 | 4.228 | **< 0.001** |
|  | Oak forest x web area | 6.7e^-4^ | 0.0045 | 367.2 | 0.127 | 0.899 |
|  | Pine forest x web area | 0.001 | 0.005 | 369.4 | 0.285 | 0.776 |

| b ) Field data (sexual traits in relationship with PHA response) | | | | | | |
| --- | --- | --- | --- | --- | --- | --- |
| Trait | Predictor | Estimate | SE | df | t-value | P - value |
| PHA response | Intercept | -0.046 | 0.008 | 17.7 | -5.196 | **< 0.001** |
|  | Oak forest | 0.081 | 0.012 | 16.6 | 6.559 | **< 0.001** |
|  | Pine forest | 0.050 | 0.012 | 16.1 | 4.067 | **< 0.001** |
|  | Tail area | 0.175 | 0.109 | 372.9 | 1.613 | 0.107 |
|  | Oak forest x tail area | -0.118 | 0.145 | 374.2 | -0.811 | 0.417 |
|  | Pine forest x tail area | -0.008 | 0.145 | 374.7 | -0.055 | 0.955 |
|  | Intercept | -0.044 | 0.009 | 19.1 | -4.920 | **< 0.001** |
|  | Oak forest | 0.081 | 0.012 | 17.7 | 6.391 | **< 0.001** |
|  | Pine forest | 0.049 | 0.012 | 16.8 | 3.931 | **0.001** |
|  | Tail depth | 0.344 | 0.183 | 370.0 | 1.876 | 0.061 |
|  | Oak forest x tail depth | -0.323 | 0.237 | 371.8 | -1.359 | 0.175 |
|  | Pine forest x tail depth | -0.082 | 0.226 | 369.9 | -0.362 | 0.717 |
|  | Intercept | -0.094 | 0.016 | 145.8 | -5.575 | **< 0.001** |
|  | Oak forest | 0.122 | 0.024 | 148.3 | 5.024 | **< 0.001** |
|  | Pine forest | 0.079 | 0.023 | 136.9 | 3.426 | **< 0.001** |
|  | Filament length | 0.278 | 0.089 | 369.1 | 3.106 | **0.002** |
|  | Oak forest x filament length | -0.236 | 0.118 | 374.7 | -1.992 | **0.047** |
|  | Pine forest x filament length | -0.168 | 0.121 | 371.3 | -1.387 | 0.166 |
|  | Intercept | -0.040 | 0.009 | 19.9 | -4.344 | **< 0.001** |
|  | Oak forest | 0.073 | 0.012 | 19.0 | 5.641 | **< 0.001** |
|  | Pine forest | 0.045 | 0.012 | 17.5 | 3.610 | **0.002** |
|  | Web area | 0.018 | 0.006 | 364.6 | 3.104 | **0.002** |
|  | Oak forest x web area | -0.012 | 0.007 | 368.5 | -1.636 | 0.102 |
|  | Pine forest x web area | -0.009 | 0.008 | 371.1 | -1.180 | 0.264 |

Table S3. The results of the models of the relationships between each relative sexual trait expression and: (a) and body condition, (b) PHA response in the mesocosms data. Sample sizes in main text.

| a ) Mesocosms data (sexual traits in relationship with body condition) | | | | | | |
| --- | --- | --- | --- | --- | --- | --- |
| Trait | Predictor | Estimate | SE | df | t-value | P - value |
| Body condition | Intercept | 0.009 | 0.015 | 179.0 | 0.568 | 0.570 |
|  | Oak forest | -0.025 | 0.022 | 179.0 | -1.119 | 0.264 |
|  | Pine forest | -0.002 | 0.023 | 179.0 | -0.116 | 0.907 |
|  | Tail area | 0.234 | 0.077 | 179.0 | 3.038 | **0.002** |
|  | Oak forest x tail area | -0.033 | 0.114 | 179.0 | -0.295 | 0.768 |
|  | Pine forest x tail area | -0.050 | 0.130 | 179.0 | -0.387 | 0.698 |
|  | Intercept | 0.017 | 0.015 | 179.0 | 1.128 | 0.260 |
|  | Oak forest | -0.032 | 0.021 | 179.0 | -1.494 | 0.137 |
|  | Pine forest | -0.012 | 0.021 | 179.0 | -0.590 | 0.555 |
|  | Tail depth | 0.647 | 0.184 | 179.0 | 3.505 | **< 0.001** |
|  | Oak forest x tail depth | -0.140 | 0.276 | 179.0 | -0.509 | 0.611 |
|  | Pine forest x tail depth | 0.448 | 0.325 | 179.0 | 1.378 | 0.169 |
|  | Intercept | 0.013 | 0.016 | 179.0 | 0.815 | 0.416 |
|  | Oak forest | -0.018 | 0.023 | 179.0 | -0.789 | 0.431 |
|  | Pine forest | -0.018 | 0.023 | 179.0 | -0.769 | 0.443 |
|  | Filament length | 0.222 | 0.236 | 179.0 | 0.938 | 0.349 |
|  | Oak forest x filament length | -0.292 | 0.332 | 179.0 | -0.880 | 0.380 |
|  | Pine forest x filament length | -0.180 | 0.316 | 179.0 | -0.569 | 0.570 |
|  | Intercept | 0.015 | 0.016 | 179.0 | 0.935 | 0.351 |
|  | Oak forest | -0.030 | 0.022 | 179.0 | -1.318 | 0.189 |
|  | Pine forest | -0.017 | 0.022 | 179.0 | -0.761 | 0.447 |
|  | Web area | 0.176 | 0.088 | 179.0 | 1.981 | **0.049** |
|  | Oak forest x web area | 0.070 | 0.125 | 179.0 | 0.564 | 0.573 |
|  | Pine forest x web area | 0.015 | 0.129 | 179.0 | 0.120 | 0.904 |

| b ) Mesocosm data (sexual traits in relationship with PHA response) | | | | | | |
| --- | --- | --- | --- | --- | --- | --- |
| Trait | Predictor | Estimate | SE | df | t-value | P - value |
| PHA response | Intercept | -0.267 | 0.046 | 5.93 | -5.750 | **0.001** |
|  | Oak forest | 0.496 | 0.066 | 6.06 | 7.508 | **<0.001** |
|  | Pine forest | 0.279 | 0.066 | 6.22 | 4.200 | **0.005** |
|  | Tail area | 0.136 | 0.138 | 178.96 | 0.989 | 0.323 |
|  | Oak forest x tail area | 0.116 | 0.205 | 178.79 | 0.565 | 0.572 |
|  | Pine forest x tail area | -0.266 | 0.230 | 177.2 | -1.158 | 0.248 |
|  | Intercept | -0.265 | 0.043 | 5.96 | -6.043 | **< 0.001** |
|  | Oak forest | 0.494 | 0.062 | 6.03 | 7.935 | **< 0.001** |
|  | Pine forest | 0.278 | 0.062 | 5.93 | 4.486 | **0.004** |
|  | Tail depth | 0.068 | 0.339 | 178.46 | 0.203 | 0.839 |
|  | Oak forest x tail depth | 0.643 | 0.507 | 178.36 | 1.269 | 0.206 |
|  | Pine forest x tail depth | -0.869 | 0.594 | 177.85 | -1.462 | 0.145 |
|  | Intercept | -0.257 | 0.048 | 6.11 | -5.256 | **0.001** |
|  | Oak forest | 0.495 | 0.069 | 6.10 | 7.148 | **< 0.001** |
|  | Pine forest | 2.777 | 0.068 | 6.01 | 4.026 | **0.006** |
|  | Filament length | 0.668 | 0.397 | 176.12 | 1.683 | 0.094 |
|  | Oak forest x filament length | -0.399 | 0.558 | 176.65 | -0.716 | 0.475 |
|  | Pine forest x filament length | -0.484 | 0.529 | 175.54 | -0.914 | 0.361 |
|  | Intercept | -0.263 | 0.044 | 6.05 | -5.866 | **0.001** |
|  | Oak forest | 0.492 | 0.063 | 6.06 | 7.767 | **< 0.001** |
|  | Pine forest | 0.284 | 0.063 | 5.98 | 4.490 | **0.004** |
|  | Web area | 0.129 | 0.153 | 173.5 | 0.849 | 0.397 |
|  | Oak forest x web area | 0.215 | 0.218 | 178.26 | 0.984 | 0.326 |
|  | Pine forest x web area | -0.033 | 0.224 | 174.88 | -0.151 | 0.880 |
